# Protective Effects of Traditional Herbal Formulas on Cisplatin-Induced Nephrotoxicity in Renal Epithelial Cells via Antioxidant and Antiapoptotic Properties

**DOI:** 10.1155/2020/5807484

**Published:** 2020-08-17

**Authors:** VinayKumar Dachuri, Phil Hyun Song, Sae-Kwang Ku, Chang-Hyun Song

**Affiliations:** ^1^Department of Anatomy and Histology, College of Korean Medicine, Daegu Haany University, Gyeongsan 38610, Republic of Korea; ^2^Research Center for Herbal Convergence on Liver Disease, Gyeongsan 38610, Republic of Korea; ^3^Department of Urology, College of Medicine, Yeungnam University, Daegu 42415, Republic of Korea

## Abstract

Acute kidney injury (AKI) is characterized by a rapid loss of renal function. Drug-induced AKI accounts for up to 60% of all cases, resulting in a severe threat particularly to hospitalized patients, but there are no effective treatments. Four polyherbal formulas, *Bojungikki-tang* (BJ), *Palmijihwang-tang* (PJ), *Oryeong-san* (OR), and *Wiryeong-tang* (WR), have long been used for treatments of symptoms of kidney disease in traditional Korean medicine. Even though they are commercially available, evidences supporting the efficacy on AKI are still lacking. Therefore, the effectiveness of polyherbs on AKI and the underlying mechanisms were examined. Renal cell damage was induced by cisplatin at 20 *μ*M and 16 *μ*M in proximal tubular epithelial cell lines of rat NRK-52E and human HK-2, respectively. The cells were treated with the polyherbal formals for 3 days, and the cell viability, antioxidant activities, and apoptosis were examined. In addition, the proliferative effects were assessed under serum-free conditions. The results were compared with those of the vehicle-treated cells as a control. Three polyherbs BJ, PJ, and WR but not OR showed strong free radical scavenging activities in the 1,1-diphenyl-2-picrylhydrazyl (DPPH) assay. The treatments of BJ, PJ, OR, and WR significantly increased the cell viabilities under cisplatin-induced nephrotoxicity. Consistent with the results of the DPPH assay, superoxide dismutase and catalase activities were increased in the cisplatin-induced cell model treated with BJ, PJ, and WR but not with OR. However, annexin-V-positive cells and cleaved caspase 3 expression were significantly reduced in the cell model treated with all of the polyherbs. Cell proliferation was observed in treatment with all of the polyherbs, which was particularly evident in the OR-treated cells. This provides effective complementary evidences to promote the development of traditional herbal formulas to treat AKI.

## 1. Introduction

Acute kidney injury (AKI) is a clinical symptom characterized by an acute loss of renal function with high morbidity and mortality (9.5% of in-hospital mortality) [1, 2]. AKI can be caused by renal ischemia-reperfusion injury, nephrotoxic damage, and sepsis. The pathogenesis involves excessive inflammatory responses, oxidative stress, and an imbalance in the renal cell injury and repair; however, specific treatment targets in the pathological process and effective therapies are still lacking [3]. The main treatments for AKI include rehydration therapy, antibiotics, dialysis, and the discontinuation of certain medicines until renal function recovers. Although AKI is a reversible condition that can be cured, it can progress to chronic kidney disease and end-stage renal disease. Given AKI is considered a global health problem, there is an urgent need to develop effective therapeutic strategies.

Drug-induced AKI accounts for up to 60% of all cases, resulting in a severe threat to hospitalized patients [4]. Cisplatin is one of the most efficient and widely used antineoplastic drugs; however, its application is restricted due to the side effects, including nephrotoxicity [5]. Indeed, the cisplatin-induced nephrotoxicity is occurred as high as 30–40% [5, 6]. The nephrotoxicity involves oxidative stress, DNA damage, mitochondrial dysfunction, caspase activation, apoptosis/necrosis, and inflammation, especially in the proximal tubules and collecting ducts [7]. Many target-based treatments including anti-inflammatory (i.e., *α*-lipoic acid and ABT-719) and antioxidant (i.e., selinium and *α*-tocopherol) reagents and others (i.e., rhIGF1, ANP, and dopamine) have been reported to be effective in both in vivo and in vitro AKI models. However, their clinical application has shown inconsistent results [8–12], which suggests that monotarget -based drugs can be not sufficient for treating AKI. Current standard cares are limited to vigorous hydration with saline and the simultaneous administration of mannitol [13]. However, patients who have progressed to end-stage renal disease have only two treatment options: dialysis or kidney transplantation. The limited treatment options for AKI have led to an increased interest in alternative therapies using traditional herbal medicine.

Asian traditional herbal medicine based on Traditional Chinese Medicine (TCM) has been widely used for the treatment of kidney disease in China, Korea, and Japan for a long time. Although several herbs containing constituents such as aristolochic acids and other plant alkaloids have been found to cause side effects, including nephrotoxicity [14], increasing evidences show that some polyherbal formulas, single herbs, and their main components have significant efficacy in AKI models via anti-inflammatory, antioxidant, or antiapoptotic activities [3, 15]. Notably, Xuebijing consisting of *Radix Paeoniae Rubra*, *Chuan dome*, *Salvia miltiorrhiza*, safflower, and Chinese angelica improves renal function in patients with scald injury-induced AKI [16]. Traditional polyherbal formulas are designed to control the balance of body functions, and the formula containing various medicinal herbs has been developed for greater synergistic and fewer side effects based on information in clinical records accumulated over time. Systems pharmacology has recently emerged as a useful tool for understanding the nature of traditional medicine formulas and their mechanisms [17, 18].

In traditional Korean medicine, *Bojungikki-tang* (BJ, *Buzhongyiqi-tang* in Chinese and *Hochuekki-to* in Japanese), *Palmijihwang-tang* (PJ, *Baweidihuang-tang* in Chinese and *Hachimijio-to* in Japanese), *Oryeong-san* (OR, *Wuling-san* in Chinese and *Gorei-san* in Japanese), and *Wiryeong-tang* (WR, *Weiling-tang* in Chinese and *Irei-to* in Japanese) are used to treat the symptoms of kidney disease [19]. Traditional pharmacology categorizes BJ for invigorating *Ki*, PJ for tonifying the kidney, and OR and WR for promoting diuresis. These polyherbal formulas were approved by the Korea Food and Drug Administration as general pharmaceuticals, and they are commercially available. Among these, OR has been reported to prevent renal injury in diabetes-induced renal dysfunction, nephrosis, and hyperuricemic models [20–22], and systems pharmacology has revealed that its mechanisms of action involve the renin-angiotensin-aldosterone system (RAAS) for treating hypertension [23]. Case reports for treatments with BJ have shown improved symptoms in patients with chronic renal failure [24, 25]. However, there have been few studies on treatments of BJ, PJ, or WR for AKI, to the best of our knowledge. Therefore, we investigated the protective effects and underlying mechanisms of action of four different polyherbal formulas on cisplatin-induced renal cell injury.

## 2. Materials and Methods

### 2.1. Preparation of Polyherbal Formulas

Four commercial polyherbs, BJ (H19™), OR (ISU™), PJ (STRECH-HWAN™), and WR (OLGA™), were used in this study. BJ, PJ, and WR were kindly provided by Hanpoong Pharmaceutical Co., Ltd. (Daejeon, Korea), and OR was provided by Jeil Pharmaceutical Co., Ltd. (Seongnam, Korea). These commercial products are approved by the Korea Food and Drug Administration, and their main herbal components are listed in Table 1. The polyherbs were dissolved in dimethyl sulfoxide (DMSO; Sigma-Aldrich, St. Louis, MO, USA) and treated in a cell medium containing 0.5% DMSO as the vehicle control. The treatment solution was filtered through a pore size of 0.22 *μ*m and stored at 4°C in the dark until use.

### 2.2. Free Radical Scavenging Activity

Antioxidant activity was assessed using 1,1-diphenyl-2-picrylhydrazyl (DPPH; Sigma-Aldrich). DPPH was prepared at 0.4 mM in methanol and mixed with each polyherb at a final concentration of 1 mg/mL. It was incubated at 37°C for 30 min in the dark, and the absorbance was measured at 517 nm using a Synergy™ H1 automated microplate reader (Bio-Tek, Winooski, VT, USA). The activity was represented as a percentage to that of distilled water containing 0.5% DMSO as a control and calculated using the following formula:

(1) $$\begin{array}{c} \text{Inhibition }\left(\%\right)=\frac{\left(\text{Absorbance of control}-\text{Absorbance of polyherb}\right)\;}{\text{Absorbance of control}}\times 100. \end{array}$$

### 2.3. Cell Culture

Two kidney proximal tubular epithelial cell lines, rat NRK-52E (NRK) and human HK-2, were obtained from the American Type Culture Collection (ATCC, URL http://www.atcc.org). The NRK and HK-2 cells were cultured in Dulbecco's modified Eagle's medium (DMEM) with high glucose (DMEM-HG; Hyclone) and Roswell Park Memorial Institute (RPMI) 1640 medium, respectively, supplemented with 10% fetal bovine serum (FBS), 100 U/ml penicillin, and 100 *μ*g/ml streptomycin (all from Gibco, Grand Island, NY, USA). The cells were grown in a 5% CO_2_ humidified incubator at 37°C. When the cells reached 80–90% confluence, they were seeded in 96- and 6-well plates at 1 × 10^4^ and 1 × 10^6^ cells/well, respectively, and starved overnight in serum-free medium before treatment.

### 2.4. Renal Cell Injury and Treatments

Renal cell injury was induced by cisplatin (Sigma-Aldrich) at concentrations of 20 *μ*M for NRK and 16 *μ*M for HK-2 as the half-maximal inhibitory concentrations (IC_50_) for 3 days. The cell model was treated with the various polyherbs, and then collected for further experiments. The treatments were carried out under serum-free conditions, and the results were compared with those of the vehicle control.

### 2.5. Cell Viability Assay

Cell viability was assessed using the 3-(4,5-dimethylthiazol-2-yl)-2,5-diphenyltetrazolium bromide (MTT) assay (TCI Chemicals, Tokyo, Japan). Cells seeded in a 96-well plate were treated with MTT at 0.5 mg/ml and incubated for 1 h at 37°C. Then, the cells were lysed using DMSO, and color development was assessed at 570 nm using a Synergy™ H1 automated microplate reader (Bio-Tek). The viability was represented as a percentage of that of the vehicle control.

### 2.6. Catalase and Superoxide Dismutase (SOD) Assay

Activities of antioxidant enzymes, catalase, and SOD were measured using their specific assay kits (#707002 and #706002, respectively, Cayman Chemicals, Ann Arbor, MI, USA), according to the manufacturer's instructions. Briefly, the cells were scraped and centrifuged at 1,000 × g for 10 min at 4°C. For catalase activity, the cell pellet was sonicated on ice in cold buffer consisting of 50 mM potassium phosphate containing 1 mM ethylenediaminetetraacetic acid (EDTA, pH 7.0) and centrifuged at 10,000 × g for 15 min at 4°C. For SOD activity, the pellet was sonicated in 20 mM HEPES buffer (pH 7.2) containing 1 mM ethylene glycol tetraacetic acid (EGTA), 210 mM mannitol, and 70 mM sucrose and centrifuged at 1,500 × g for 5 min at 4°C. The resultant pellets were reacted with formaldehyde for catalase and tetrazolium salt for SOD; they were then assessed at 540 nm and 450 nm, respectively, using a microplate reader, under the standard curves.

### 2.7. Flow Cytometry Analysis

Cells were collected using Accutase (STEMCELL Technologies Inc., Vancouver, Canada), and their apoptosis and proliferation were measured. Apoptotic cells were measured using annexin-V-fluorescein isothiocyanate (FITC)/propidium iodide (PI) staining. Briefly, the prepared cells were stained with an anti-annexin-V-FITC antibody (Abcam, Cambridge, UK, dilution of 1 : 1000) for 30 min and then with PI (Life Technologies, Carlsbad, CA, USA) at 50 *μ*g/ml for 10 min. For proliferative cell measures, the cells were fixed in cold methanol for 10 min, and the cell membranes were permeated by treatment with saponin (TCI Chemicals) at 1 mg/ml for 30 min. The cells were incubated with a rabbit anti-Ki67 antibody (Abcam, dilution of 1 : 100) for 30 min and then with an Alexa 488-conjugated goat anti-rabbit IgG antibody (Life Technologies, dilution of 1 : 1000). All steps were performed on ice, and the cells were washed five times with phosphate-buffered saline (PBS) containing 2% FBS between each step. Cell fluorescence was analyzed using a BD Accuri C6 Plus flow cytometer (BD Bioscience, San Jose, CA, USA).

### 2.8. Western Blot Analysis

Cells were scraped and centrifuged at 10,000 × g for 10 min at 4°C. The cell pellet was lysed in radioimmunoprecipitation assay (RIPA) buffer (PA 19464, Rock Land, Pottstown, PA, USA) containing 1 mg/ml protease inhibitor (leupeptin, Roche, Mannheim, Germany) for 30 min on ice. After centrifuging at 10,000 × g for 10 min at 4°C, the supernatant was measured for total protein contents using the BCA assay (Thermo Fisher, Rockford, IL, USA); then, the samples were mixed with a loading buffer (Bio-Rad, Hercules, CA, USA) and subsequently boiled for 10 min. The samples containing equal amounts of protein were electrophoresed through 12% SDS-PAGE gels and electrotransferred onto a nitrocellulose membrane using semidry blot transfer (Bio-Rad). After blocking with 5% skim-milk in Tris-buffered saline plus Tween (5% TBST, Bio-Rad), the membrane was incubated with mouse anticleaved caspase-3 (Cell Signaling, Danvers, MA, USA: dilution of 1 : 100) and anti-*β*-actin antibodies (Abcam, dilution of 1 : 2,000) in 1% TBST at 4°C overnight. Next day, it was incubated with a horseradish peroxidase- (HRP-) conjugated anti-mouse secondary antibody (Bio-Rad, dilution of 1 : 1000) for 1 h. The membrane was washed five times with TBST for 30 min after incubation with primary and secondary antibodies. The expressions were visualized using an enhanced chemiluminescence substrate (WESTAR*η*C2.0, Cyanagen, Bologna, Italy) and analyzed using a Bio-Rad ChemiDoc instrument (Bio-Rad).

### 2.9. Statistical Analysis

The data are expressed as the mean and standard deviation (SD). Since there were no significances in the Leven test for the homogeneity of variance, the results were analyzed by one-way analysis of variance (ANOVA), followed by Tukey's post hoc tests. Each experiment was performed at least three times. A *p* value less than 0.05 was considered statistically significant.

## 3. Results

### 3.1. Antioxidant Properties of Polyherbal Formulas

**Figure 1:**
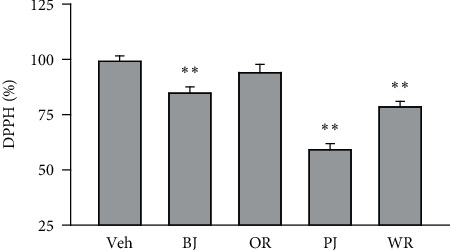
Free radical scavenging activity of polyherbal formulas. Antioxidant activity of polyherbal formulas, *Bojungikki-tang* (BJ), *Oryeong-san* (OR), *Palmijihwang-tang* (PJ), and *Wiryeong-tang* (WR), was assessed using the 1,1-diphenyl-2-picrylhydrazyl (DPPH) assay. The results were compared with distilled water containing 0.5% dimethyl sulfoxide (DMSO) as the control, and values are represented as a percentage of the control. ^*∗∗*^*p* < 0.01 versus the control.

Free radical scavenging activity of the polyherbal formulas was assessed using DPPH reagent, and the activity was compared with that of the 0.5% DMSO vehicle control (Figure 1). One-way ANOVA showed significant main effects for the group (*F* = 36.3, *p* < 0.01). The post hoc test versus the vehicle control revealed significant decreases by 15.3% in BJ, 40.9% in PJ, and 21.5% in WR (*p* < 0.05). The activity was a little reduced by 5.9% in OR; however, it was not significant.

### 3.2. Protective Effects of Polyherbal Formulas in Cisplatin-Induced Renal Cell Injury

**Figure 2:**
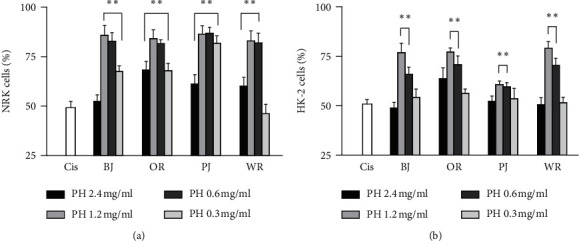
Protective effects of polyherbal formulas in cisplatin-induced renal cell injury. Renal cell injury was induced by cisplatin in rat NRK (a) and human HK-2 (b). The cell model was coincubated with BJ, OR, PJ, and WR at 0.3–2.4 mg/kg for 3 days. Values are represented as a percentage to the cisplatin nontreated normal group. ^*∗∗*^*p* < 0.01 versus the cisplatin-treated vehicle control (Cis).

In the cisplatin-induced renal cell injury model, the cell viabilities of the vehicle negative control showed IC_50_ : 49.1% in NRK and 50.8% in HK-2, compared with those of cisplatin nontreated cells (Figure 2). One-way ANOVA showed significant main effects for the group in NRK (*F* = 62.4, *p* < 0.01) and HK-2 (*F* = 11.9, *p* < 0.01). The post-hoc tests versus the negative control showed significant increases in treatments with BJ at 0.3–1.2 mg/ml, OR and PJ at 0.3–2.4 mg/ml, and WR at 0.6–2.4 mg/ml in NRK, and in treatments with BJ, OR, PJ, and WR at 0.6 and 1.2 mg/ml in HK-2 (*p* < 0.05). The viabilities seemed to be evidently increased in the treatment at a concentration between 0.6 and 1.2 mg/ml. The increased ratios to the negative control were 1.7-folds in treatments with BJ, OR, and PJ and 1.8-folds in WR at 1.2 mg/ml in NRK, while they were 1.5-folds in treatments with BJ and OR and 1.2- and 1.6-folds in treatments with PJ and WR, respectively, in HK-2.

### 3.3. Enhanced Activities of Antioxidant Enzymes in Cisplatin-Induced Renal Cell Injury

**Figure 3:**
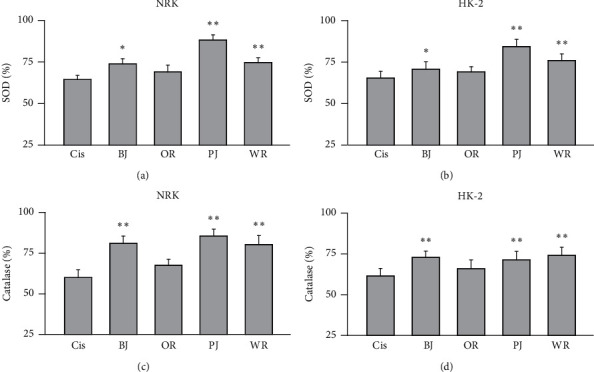
Antioxidant effects of polyherbal formulas in cisplatin-induced renal cell injury. The cisplatin renal cell injury model was treated with BJ, OR, PJ, and WR at 1 mg/ml for 3 days. The activities of superoxide dismutase (SOD (a, b) and catalase (c, d) were assessed. Values are represented as a percentage to the cisplatin nontreated group. ^*∗∗*^*p* < 0.01 and ^*∗*^*p* < 0.05 versus the cisplatin-treated vehicle control (Cis).

The negative control in the renal cell injury model versus cisplatin nontreated cell showed reduced activities of SOD and catalase by 36.3% and 35.4%, respectively, in NRK and by 40.8% and 39.4% in HK-2 (Figure 3). There were significant differences among the groups for SOD level in NRK (*F* = 105.5, *p* < 0.01) and HK-2 (*F* = 157.2, *p* < 0.01) and for catalase in NRK (*F* = 80.9, *p* < 0.01) and HK-2 (*F* = 84.6, *p* < 0.01). Consistent with the results of the DPPH assay, the post hoc tests versus the negative control revealed significant increases in the levels of SOD and catalase of NRK and HK-2 treated with BJ, PJ, and WR (*p* < 0.05) but not for OR. The increased levels of SOD were 1.2-, 1.4-, and 1.2-folds in treatments with BJ, PJ, and WR, respectively, in NRK, and they were 1.1-, 1.3-, and 1.2-folds in HK-2. The increased levels of catalase were 1.4-, 1.4-, and 1.3-folds in NRK, and they were 1.2-folds in BJ, PJ and WR in HK-2.

### 3.4. Antiapoptotic Effect of Polyherbal Formulas in Cisplatin-Induced Renal Cell Injury

**Figure 4:**
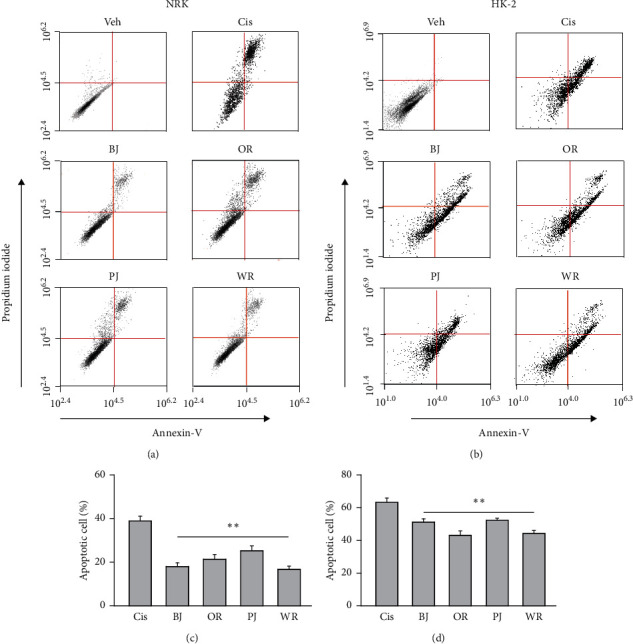
Flow cytometry analysis on apoptosis in cisplatin-induced renal cell injury. The renal cell injury model was treated with BJ, OR, PJ, and WR at 1 mg/ml for 3 days, and the apoptotic cells were measured using staining with annexin-V/propidium iodide (a, b). The results are represented as a percentage to the cisplatin nontreated group (Veh) in NRK (c) and HK-2 (d). ^*∗∗*^*p* < 0.01 versus the cisplatin-treated vehicle control (Cis).

**Figure 5:**
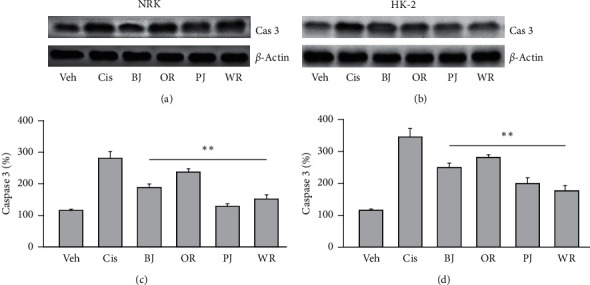
Antiapoptotic effects of polyherbal formulas in cisplatin-induced renal cell injury. The renal cell injury model was treated with BJ, OR, PJ, and WR at 1 mg/ml for 3 days, and the expression of cleaved caspase 3 (Cas3) was assessed by Western blotting (a, b). The expression was normalized by the *β*-actin levels. Values are represented as a percentage to the cisplatin nontreated group (Veh) in NRK (c) and HK-2 (d). ^*∗∗*^*p* < 0.01 versus the cisplatin-treated vehicle control (Cis).

Annexin-V-positive apoptotic cells were few in cisplatin nontreated NRK and HK-2, while they were increased in the negative control of the renal cell injury model (Figure 4). However, they trended to be reduced in NRK and HK-2 treated with the polyherbal formulas. There were significant main effects for the group in NRK (*F* = 115.4, *p* < 0.01) and HK-2 (*F* = 588.4, *p* < 0.01). The post hoc tests versus the negative control revealed significant decreases in the apoptotic cells of NRK and HK-2 treated with BJ, OR, PJ, and WR (*p* < 0.05). The reduced ratios were 2.2-, 1.8-, 1.5-, and 2.3-folds in NRK treated with BJ, OR, PJ, and WR, respectively, and they were 1.2-, 1.5-, 1.2-, and 1.4-folds in HK-2. There were no significant differences in the early apoptotic cells (only annexin-V positive) or apoptotic cell death (annexin-V/PI double positive) between the negative control and treatment groups with polyherbal formulas. Similarly, the expression of caspase 3 was increased in the negative control of the renal cell injury model compared with cisplatin nontreated NRK and HK-2, while it was reduced in the treatment with the polyherbal formulas (Figure 5). There were significant differences among groups in NRK (*F* = 160.7, *p* < 0.01) and HK-2 (*F* = 144.4, *p* < 0.01). The post hoc tests versus the negative control revealed significant decreases in the expression of caspase 3 in NRK and HK-2 treated with BJ, OR, PJ, and WR (*p* < 0.01). The reduced ratios were 1.5-, 2.2-, 1.2-, and 1.9-folds in NRK treated with BJ, OR, PJ, and WR, respectively, and they were 1.4-, 1.7-, 1.3-, and 2.0-folds in HK-2.

### 3.5. Proliferative Effects of Polyherbal Formulas on Renal Tubular Epithelial Cells

**Figure 6:**
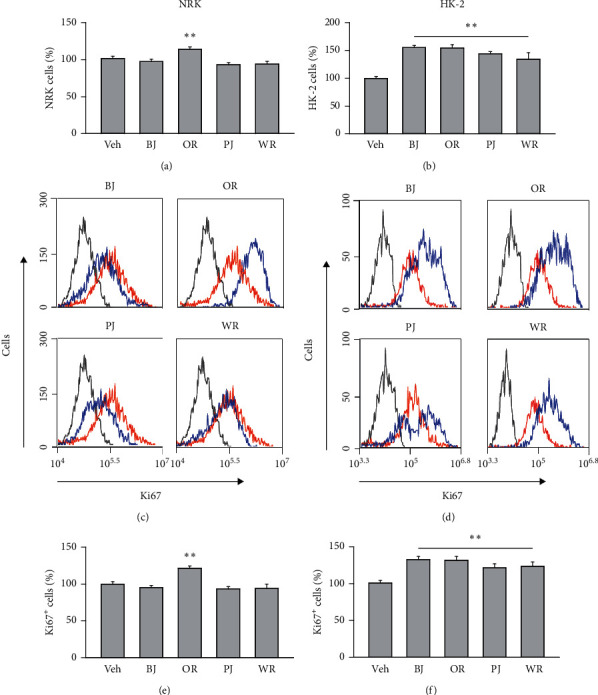
Proliferative effects of polyherbal formulas in renal epithelial cells. NRK and HK-2 were treated with BJ, OR, PJ, and WR at 1 mg/ml under serum-free conditions for 3 days, and the viabilities were assessed (a, b). The Ki67-positive cells were measured by flow cytometry (c, d). Blue and red lines indicate the polyherb treatment and vehicle control (Veh), respectively. The black is for experimental control omitting primary antibody in staining. Values are represented as a percentage of the Veh. ^*∗∗*^*p* < 0.01 versus the vehicle.

NRK and HK-2 cells were treated with the polyherbal formulas at 1 mg/ml under serum-free conditions for 3 days, and the cell growth was assessed (Figure 6). Comparing to the vehicle treatment, the cells were significantly increased by 1.1-folds in NRK treated with OR and by 1.6-, 1.5-, 1.4- and 1.3-folds in HK-2 treated with BJ, OR, PJ, and WR, respectively (*p* < 0.01). Consistently, the Ki67-positive cells were observed more in NRK treated with OR and in HK-2 treated with BJ, OR, PJ, and WR. There were significant differences among the groups in NRK (*F* = 15.4, *p* < 0.01) and HK-2 (*F* = 23.1, *p* < 0.01). The Ki67-positive cells were significantly increased by 1.2-folds in NRK with OR and by 1.3-folds with BJ and OR and by 1.2-folds in HK-2 with PJ and WR, as compared to the vehicle group (*p* < 0.01).

## 4. Discussion

In this study, we showed that four traditional polyherbal formulas potentially prevented cisplatin-induced renal cell injury by inhibiting oxidative stress and apoptotic cell death and enhancing cellular proliferation. Traditional polyherbal formulas consisting of natural products have advantages over chemical compound-based drugs, including less side effects, variable bioavailability, and vigorous biological activity. Many traditional Korean medicine-based polyherbs are commercially available because accumulated clinical results prove that they have been effective for a long time. However, scientific evidence is still needed to clarify their efficacy and therapeutic mechanisms. We have screened potentials of anti-AKI polyherbal formulas with multitargeting to complement the treatment limitations of monotargeted agents. Considering that a goal of drug discovery is developing optimal combinations consisting of effective compounds for treating diseases, verifying the efficacy in traditional polyherbal formulas already proven clinically can be the most efficient and fastest strategy for novel drug development in AKI treatment.

Many medicinal herbs have been reported to improve the nephrotoxicity of AKI via antioxidant and anti-inflammatory properties [3, 15]. Cisplatin produces excessive reactive oxygen species (ROS) and induces mitochondrial dysfunction and macrophage activation, which further increases ROS release and decreases the cellular antioxidant defense system [6]. Since the process promotes renal neutrophil infiltration and tubular cell apoptosis, the polyherbs of BJ, PJ, and WR showing strong free radical scavenging activities and enhanced levels of SOD and catalase activities might reduce annexin-V-positive cells and activation of caspase 3 that plays a key role in apoptosis. Actually, the deletion of extracellular SOD3 has been shown to lead to functional deterioration in AKI [26]. There are concerns that the antioxidant activity of polyherbal formulas might compromise the antitumor activity of cisplatin. However, oxidative stress can increase cisplatin-resistance, and antioxidant reagents can also sensitize tumor cell to cisplatin [27]. Conversely, even though OR showed no antioxidant properties, it inhibited apoptosis and prevented cisplatin-induced cell death. Furthermore, while BJ, PJ, and WR showed cellular proliferative effects, those of OR were particularly prominent. The fact that OR promoted the cell growth under serum-free conditions for 3 days is interesting because it suggests potential regenerative effects in the kidney. There have been few reports involving the renal signaling pathways; one study has shown that OR has nephroprotective effects by suppressing the activation of the toll-like receptor 4/myeloid differentiation factor 88 signaling and pyrin domain containing 3 inflammasome in hyperuricemic mice [20]. However, the underlying mechanism is completely uncertain in this study, and we are planning the future study to clarify the signaling pathways related to antioxidant and proliferative properties.

Many studies have shown efficacy of individual herbs and their main compounds on AKI models and the relevant mechanisms such as antioxidant, anti-inflammatory, and antiapoptotic effects [15]. The effective single herbs on AKI include *Astragalus* root, ginseng, and *Glycyrrhiza*, which are constituents of the BJ formula; Hoelen in PJ, OR, and WR; moutan root bark and *Cornus* fruit in PJ; and *Peony* root and *Magnolia* bark in WR. TCM-based polyherbal formulas also showed preventive effects on AKI [15]. For instance, the combination of *Angelica gigas* and *Astragalus* roots, as the herbal components of BJ, improves renal blood flow by increasing nitric oxide production [28], and *Zhibai Dihuang Wan*, which included the same four herbal components as PJ, attenuates renal injury and apoptosis [29]. Given that the polyherbal formula consists of several medicinal herbs containing hundreds of compounds, the mechanism studies on the multiple targets are difficult to analyze. However, traditional polyherbal formulas can be a useful source of novel herbal combinations for the treatment of AKI from a different perspective. For example, OR is used traditionally as diuretic, and its main herbal component, *Alimatis rhizoma*, has been currently reported to have remarkable diuretic activity [30]. The systems pharmacology approach has demonstrated the improvement of hypertension by the activation of the RAAS [23]. It is expected that multitargeting of the five herbal components of OR may have synergistic effects of diuretic activity coupled with antiapoptotic and proliferating effects in protecting against cisplatin-induced AKI.

The clinical application of traditional herbal medicines in patients with kidney disease is rarely recommended because of previous reports on potential nephrotoxicity [14]. However, the present study showed the protective effects of BJ, PJ, OR, and WR on cisplatin-induced renal cell toxicity. Furthermore, some studies have shown the favourable effects of some of these polyherbal formulas in cancer: BJ restores the immune system in end-stage cancer [31], and OR ameliorates nephrotic syndrome induced by the antitumor drug, Adriamycin [32]. Traditional herbal formulas provide enormous resources for discovering novel medicinal combinations for treating AKI. This study clearly demonstrated that the polyherbal formulas of BJ, PJ, OR, and WR protected against cisplatin-induced renal cell injury via antioxidant, antiapoptotic, and proliferative effects.

## Figures and Tables

**Table 1:** Medicinal herbs consisting of polyherbal formulas used.

	Herbal components
BJ	*Angelica gigas* root (0.6 g), *Astragalus* root (1.9 g), *Atractylodes* rhizome (R.) white (1.3 g), *Bupleurum* root (0.4 g), *Cimicifuga R*. (0.4 g), *Citrus unshiu* peel (0.6 g), ginseng (1.3 g), and *Glycyrrhiza* (1.3 g)
OR	*Alisma* R. (1.7 g), *Atractylodes* R. (1.0 g), Cinnamomi cortex (0.7 g), Chuling (1.0 g), and Hoelen (1.0 g)
PJ	*Alisma* R. (11.1 mg), Cinnamomi Ramulus (3.7 mg), *Cornus* fruit (14.8 mg), *Dioscorea* R. (14.8 mg), Hoelen (11.1 mg), moutan root bark (11.1 mg), Pulvis aconiti tuberis purificatum (3.7 mg), and steamed *Rehmannia* root (29.7 mg)
WR	*Alisma* R. (1.0 g), AR (1.0 g), *Atractylodes* R. white (1.0 g), Chuling (1.0 g), *Cinnamomi ramulus* (0.8 g), *Citrus unshiu* peel (1.0 g), Ginger (0.7 g), *Glycyrrhiza* (0.7 g), Hoelen (1.0 g), jujube (1.0 g), *Magnolia* bark (1.0 g), and peony root (1.0 g)

Amounts of main herbal components for *Bojungikki-tang* (BJ), *Oryeong-san* (OR), *Palmijihwang-tang* (PJ), and *Wiryeong-tang* (WR) are indicated.

## Data Availability

The data used to support the findings of this study are available from the corresponding author upon request.
